# The FRAME-IS: a framework for documenting modifications to implementation strategies in healthcare

**DOI:** 10.1186/s13012-021-01105-3

**Published:** 2021-04-07

**Authors:** Christopher J. Miller, Miya L. Barnett, Ana A. Baumann, Cassidy A. Gutner, Shannon Wiltsey-Stirman

**Affiliations:** 1 VA Boston Healthcare System, Center for Healthcare Organization and Implementation Research (CHOIR), Boston, MA USA; 2 Department of Psychiatry, Harvard Medical School, Boston, MA USA; 3 Department of Counseling, Clinical, & School Psychology, University of California, Santa Barbara, Santa Barbara, CA USA; 4 Washington University at St. Louis, St. Louis, MO USA; 5 ViiV Healthcare, Innovation & Implementation Science, Research Triangle, NC USA; 6 Department of Psychiatry, Boston University School of Medicine, Boston, MA USA; 7 National Center for PTSD Dissemination and Training Division, Palo Alto, CA USA; 8 Department of Psychiatry and Behavioral Sciences, Stanford University, Stanford, CA USA

**Keywords:** Modification, Adaptation, Implementation strategy

## Abstract

**Background:**

Implementation strategies are necessary to ensure that evidence-based practices are successfully incorporated into routine clinical practice. Such strategies, however, are frequently modified to fit local populations, settings, and contexts. While such modifications can be crucial to implementation success, the literature on documenting and evaluating them is virtually nonexistent. In this paper, we therefore describe the development of a new framework for documenting modifications to implementation strategies.

**Discussion:**

We employed a multifaceted approach to developing the Framework for Reporting Adaptations and Modifications to Evidence-based Implementation Strategies (FRAME-IS), incorporating multiple stakeholder perspectives. Development steps included presentations of initial versions of the FRAME-IS to solicit structured feedback from individual implementation scientists (“think-aloud” exercises) and larger, international groups of researchers. The FRAME-IS includes core and supplementary modules to document modifications to implementation strategies: what is modified, the nature of the modification (including the relationship to core elements or functions), the primary goal and rationale for the modification, timing of the modification, participants in the modification decision-making process, and how widespread the modification is. We provide an example of application of the FRAME-IS to an implementation project and provide guidance on how it may be used in future work.

**Conclusion:**

Increasing attention is being given to modifications to evidence-based practices, but little work has investigated modifications to the implementation strategies used to implement such practices. To fill this gap, the FRAME-IS is meant to be a flexible, practical tool for documenting modifications to implementation strategies. Its use may help illuminate the pivotal processes and mechanisms by which implementation strategies exert their effects.

**Supplementary Information:**

The online version contains supplementary material available at 10.1186/s13012-021-01105-3.

Contributions to the literature

- Implementation strategies are typically required to ensure that evidence-based practices (EBPs) are adopted in real-world clinical settings.
- These implementation strategies, like EBPs themselves, are often modified based on the clinical setting or patient population being served.
- To our knowledge, the field lacks a systematic way of documenting and tracking modifications to implementation strategies, complicating efforts to understand the mechanisms by which those implementation strategies exert their effects.
- To fill this gap, in this paper, we describe the development of the Framework for Reporting Adaptations and Modifications to Evidence-based Implementation Strategies (FRAME-IS).
- Systematic use of the FRAME-IS will allow healthcare system leaders, administrators, and implementation scientists to better understand the ways that implementation strategies may be modified to improve health outcomes across settings and populations.

## Introduction

Implementation science aims to maximize the adoption, use, and sustainability of evidence-based practices (EBPs) in real-world healthcare settings. These EBPs can include specific medications, treatment algorithms, manualized therapies, and ways of structuring care. Adaptation of an EBP has been defined as the deliberate alteration of its design or delivery to improve its fit in a given context [1, 2]. For example, an evidence-based psychotherapy that is traditionally completed in 12 sessions could be shortened for use in a new clinical setting if 12 sessions is shown to be burdensome, too costly, or unnecessary for that patient population. Modifications to EBPs may be made in either an ad hoc or planful manner; the latter category of planned modifications may be labeled as adaptations [3, 4]. Despite the importance of modifications for improving EBP effectiveness [5] and decreasing healthcare disparities [6], they remain understudied in implementation science. Recent work has aimed to fill this gap by fleshing out ways to design, document, and evaluate modifications to EBPs (e.g., [7–10]).

It is increasingly evident that similar processes of tailoring and modification occur for implementation strategies as they are used to support implementation of EBPs in different contexts. Implementation strategies are methods or techniques used to adopt, implement, and sustain EBPs in routine practice [11]. Implementation strategies range from relatively “light touches” (e.g., audit and feedback [12]) to more intensive strategies that include multiple components and may act on more than one level of a health system (e.g., implementation facilitation [13]). Recent work suggests little consensus in the field regarding the selection of specific implementation strategies for a given implementation project, or the mechanisms of action for those strategies [14]. As such, scholars have suggested that implementation strategies should be theoretically or empirically driven, and their components (e.g., actions, actors, goals) described so as to promote replicability and testing of their mechanisms of action [15–17].

Concurrently, scholars acknowledge the importance of modifying implementation strategies to fit with the unique characteristics of the EBP, setting, and stakeholders involved in an implementation effort [18]. For example, consider provider training as an implementation strategy. A traditional training may involve in-person workshops, but due to the COVID-19 pandemic, many trainings have been adapted to be conducted online. In addition to tracking the specific implementation strategies being used in a given project [19, 20], we advocate for the need to document and evaluate *modifications* to implementation strategies that have been well-defined and characterized. Although implementation strategies should always be operationalized to fit each context, documentation and reporting of these adaptations has to date not been consistently undertaken. It is crucially important to develop a more nuanced understanding of modifications to implementation strategies because of the inherent dynamism and complexity of implementation itself [5]. Without such an understanding, it is difficult to determine the processes or mechanisms by which implementation strategies exert their effects on implementation outcomes. Furthermore, because many implementation strategies may consist of several components delivered in dynamic contexts (e.g., [19]), changes are typically required to one or more of those components to improve fit or address constraints. Indeed, the modification of implementation strategies fits within co-creation models, in which researchers and stakeholders collaborate to exchange, generate, and utilize knowledge [21–23]. Local modifications are key to co-creation models [24], which provide opportunities to generate collaborative knowledge about how modifications to implementation strategies impact clinical and implementation outcomes.

A first step for understanding modifications to implementation strategies is to develop a framework for characterizing those modifications across studies and settings. This can allow implementation scientists, healthcare leaders, and quality improvement specialists to track when, why, and how implementation strategies have been modified. Akin to developing a database of modifications to EBPs [25], we propose that systematic documentation is needed to determine what modifications to implementation strategies are associated with successful versus unsuccessful implementation.

Based on our previous work on developing a framework for tracking adaptations of EBPs, we have used the Framework for Reporting Adaptations and Modifications – Expanded (FRAME) [10] as a foundation from which to build a new framework, focusing on modifications that may be made to implementation strategies rather than EBPs. As described in more detail below, we found that a new framework was necessary because our pilot work highlighted key differences in tracking modifications to implementation strategies versus EBPs. Thus, the Framework for Reporting Adaptations and Modifications to Evidence-based Implementation Strategies (FRAME-IS) aims to meet the need in the implementation science field for a tool to document modifications to implementation strategies. Below, we describe the development of the FRAME-IS and include an example of its application to a recent implementation trial.

## Development of the FRAME-IS

The FRAME-IS is based on the FRAME, the development of which is described in detail elsewhere [10, 26] Briefly, the FRAME is a framework for tracking modifications to EBPs focused on *what* to adapt, alongside a related adaptation framework blending the *content* and *processes* of adaptation [27, 28]. The FRAME was developed based on earlier research [26], combined with results from a literature review, focus groups, and coding process rooted in grounded theory [29, 30].

Our process for in turn developing the FRAME-IS can be found in Table 1. We began with the FRAME [10] and used it to develop a first draft of the FRAME-IS by changing the wording to refer to modifications to implementation strategies instead of EBPs. We also changed the language regarding the personnel involved in the modification (e.g., emphasizing the role of implementers themselves in the modification process). The authors then used the preliminary version of the FRAME-IS to describe modifications to implementation strategies in their own ongoing and completed projects, which informed further refinement (e.g., [31]). While the FRAME-IS is meant to be applicable to implementation efforts in traditional healthcare settings, it also includes language related to educational settings where some healthcare interventions may be implemented.

**Table 1:** FRAME-IS development process

Steps	Process/operationalization
1. Identify goal and scope	- Goal: to develop a framework for documenting modifications to implementation strategies that includes the rationale for those modifications as well as the personnel involved in the modification decision. - Scope: implementation strategies meant to enhance the uptake of evidence-based practices in healthcare and educational settings.
2. Identify changes needed from previous framework	The first author (CJM) reviewed the FRAME and identified areas where language would need to be changed to refer to modifications to implementation strategies rather than modifications to evidence-based practices.
3. Develop draft of new framework	The first author (CJM) developed the initial version of FRAME-IS; this version was reviewed and edited by all co-authors.
4. Pilot new framework	Study authors piloted the initial version of FRAME-IS in their own ongoing implementation studies and made additional edits.
5. Solicit and Integrate stakeholder feedback	- Presented draft FRAME-IS to several groups of stakeholders, including international groups of implementation scientists and practitioners. Sought broad feedback on content, format, and structure of the draft FRAME-IS. - Conduced one-on-one “think-alouds” with implementation experts (*n* = 6) to provide further refinement; integrated various sources of feedback to develop final FRAME-IS.
6. Apply new framework to additional project(s)	Applied the final FRAME-IS to an ongoing study by one study author (MB; see text and Table 2 for details).

We then presented the FRAME-IS to several groups of stakeholders, including implementation experts from our networks, as well as international groups of implementation scientists and practitioners. These groups included the Washington University Network for Dissemination and Implementation Researchers (WUNDIR [32];) and the VA Quality Enhancement Research Initiative Implementation Research Group (QUERI IRG [33];). During these presentations, stakeholders (total *n* > 50) noted the importance of streamlining the FRAME-IS (e.g., eliminating some options, emphasizing a modular approach). We also used those presentations to identify volunteers to complete one-on-one “think-aloud” sessions [34]. Thinking aloud requires stakeholders to talk aloud while performing a task, allowing developers to understand their experiences and perspectives to shape the product to better meet their needs and constraints. This method has been applied in research on cognitive processes and in human-centered design approaches to product development. In our case, think-aloud participants (*n* = 6) were asked to review the FRAME-IS, apply it to implementation strategies while verbalizing their thought process as they did so, and provide general feedback afterward. The authors facilitated this process and took notes, which were reviewed by the full author team and used to refine the FRAME-IS. These sessions took about 30 min to complete and focused on a variety of implementation strategies including implementation facilitation, provider training, and audit/feedback. Aggregate results suggested the need for additional streamlining and clarification of the answer options to be included in the FRAME-IS. Stakeholders also suggested that the module documenting the rationale for the modification (Module 4 below) should be tied more closely to existing theories and frameworks (e.g., [16, 35]). Incorporating these suggested revisions led to the final version, which consists of a series of modules meant to capture different aspects of the modification process. The resulting FRAME-IS is multifaceted and is intended to be applicable to a variety of implementation strategies and contexts. Below, we describe each component of the FRAME-IS, as well as literature relevant to its conceptualization and challenges that may emerge in its use.

## Components of the FRAME-IS

**Fig. 1:**
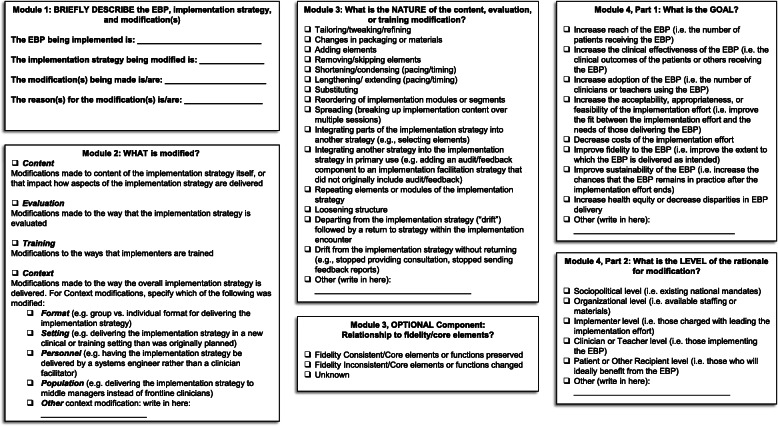
The FRAME-IS (core modules)

**Fig. 2:**
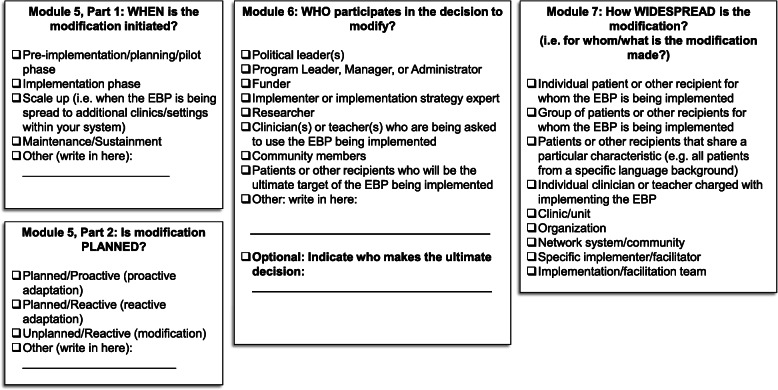
The FRAME-IS (optional modules)

Based on stakeholder feedback, the FRAME-IS is modular and includes both core (i.e., required) and optional modules to maximize its practicality across implementation projects with a variety of goals, priorities, and available resources. Core modules can be found in Fig. 1, and optional modules can be found in Fig. 2. The decision regarding which modules to designate as core versus optional was made by the authors based on consensus discussions, incorporating stakeholder feedback. Additional descriptive text can be found in the supplement. Completing core modules requires specifying: a brief description of the EBP, implementation strategy, and modifications (Module 1); what is modified (Module 2); the nature of the modification (content, evaluation, or training modifications only; Module 3); and the rationale for the modification (Module 4). Optional modules, which can be completed at the discretion of the study investigators or project team, involve specifying when the modification occurred, and whether it was planned (Module 5); who participated in the decision to modify (Module 6); and how widespread the modification is (Module 7). Module 3, while itself considered a core module, includes the option of documenting the extent to which the modification was considered fidelity-consistent with the original implementation strategy.

### Module 1: Brief description of the EBP, implementation strategy, and modification(s)

To facilitate tracking modifications and to complete the remainder of the FRAME-IS, we recommend briefly describing the EBP in question, and the initially defined implementation strategy. The Expert Recommendations for Implementing Change (ERIC) compilation [11] may be useful in describing the implementation strategy. If it is feasible, more comprehensive guidance [16] may be used to describe the implementation strategy in more detail to delineate the core elements, processes, or functions of the strategy. We also suggest identifying potential initial modifications to the strategy. We note that many modifications may be “bundled”—i.e., may involve changes to multiple aspects of the implementation strategy. For example, the content and the length of a provider training may be modified simultaneously. In those cases, the research or implementation team can determine whether to complete the FRAME-IS separately for each modification or to document all of the separate modifications at once. Completing it separately for each individual modification may allow for finer-grained analysis of what was most helpful—but may also represent an undue documentation burden in cases where separate components of a modification cannot be disentangled. Similarly, depending on the goal of the project, users should determine whether it is best to use the FRAME-IS separately for each component of a multifaceted implementation strategy or to define the entire strategy and modification in Module 1. For example, Leadership and Organizational Change for Implementation (LOCI [36]) is a multi-component implementation strategy that includes coaching calls. If the modification in question involved changing the frequency of LOCI coaching calls, the research team would need to decide whether the change in coaching call frequency should be documented alongside modifications that may be made simultaneously to other LOCI components.

### Module 2: What is modified?

The FRAME-IS includes four broad categories of modifications to implementation strategies: Content, Context, Evaluation, and Training. These four categories mirror those included in the FRAME, but with some key distinctions. A Context modification refers to changes to the setting or the way the implementation strategy is delivered. For example, if the implementation strategy being modified was implementation facilitation, the context could change if facilitation was provided virtually as opposed to in person. We note that there are some distinctions in Content and Training modifications to implementation strategies. For example, if the implementation strategy in question is a *clinician* training workshop, then modifications to the content of the workshop itself would qualify as a Content modification (because it is part of the package of implementation strategies). In contrast, changes to how *implementers* are trained—e.g., by modifying the ways that external facilitators are trained in the context of implementation facilitation—would qualify as a Training modification. Modifications to evaluation refer to changes in the way that an implementation strategy is evaluated. For example, recent work has aimed to uncover the “core activities” of implementation facilitation [37]; ongoing studies are incorporating assessments of these core activities into their implementation evaluation plans (e.g., [38]).

### Module 3: What is the nature of the content, evaluation, or training modification?

For Content, Evaluation, and Training modifications, it is important to track the nature of the modification itself. These modifications can range from relatively small tweaks or more sweeping changes to the implementation strategy—or even abandonment of an implementation strategy or component altogether. The elements of the FRAME-IS in this domain closely mirror those of the FRAME, but with language specifying modifications made to an implementation strategy rather than an EBP. Adding or removing/skipping elements refer to specific aspects of a discrete implementation strategy (e.g., removing role plays from a training workshop) or package of strategies (e.g., removing feedback, adding incentives in a leadership program). Pacing may refer to pacing of training or frequency of feedback. Integration refers to incorporation of other implementation strategies or approaches (e.g., adding feedback or incentives to training and supervision when the first round of implementation suggests that results are not as robust as desired). Loosening structure might include coaching “on the fly” or immediately after a clinical interaction rather than at scheduled times. Substituting may include adaptations such as changing fidelity assessment from observer-rated to self-report.

As an optional portion of this module, practitioners or researchers may also be interested in documenting the extent to which the modification was conducted with fidelity [39]. In this context, fidelity may be defined as “the adherence to the intervention components, competence with which the intervention is delivered, and differentiation from other treatments” [1]. Fidelity-consistent modifications may be expected to have better outcomes than fidelity-inconsistent ones, which may represent “drift” as they remove core elements of the strategy. Core elements or functions, in this context, refer to components or topics considered essential to that implementation strategy [39]. For example, removing the feedback component from an audit and feedback implementation strategy would likely be fidelity-inconsistent. We acknowledge, though, that the relationships among fidelity, adaptation, and outcomes are complex and not fully understood [40]. Fundamental questions regarding balancing adaptation and fidelity have yet to be answered—especially for the substantial proportion of implementation strategies that are meant to be inherently adaptable, flexible, or modular (e.g., [13]). Thus, completion of this portion of the module may not be warranted in all cases.

### Module 4: What is the rationale for the modification?

The goal of this module is to document why a given modification was made to a given implementation strategy. This may allow interested parties to determine what rationales are associated with more or less successful modifications. We break the rationale into two components. First, regarding the goal of the modification, we have derived answer options in part from the Reach – Effectiveness – Adoption – Implementation – Maintenance ((RE-AIM) Framework [35] and Proctor’s implementation outcomes [15], plus one option related to health equity. We note that some goals may be related to the implementation effort itself, while others may be more directly related to the EBP being implemented. We also note that many modifications may aim to achieve multiple goals. For example, shortening an EBP training may simultaneously reduce costs, increase adoption, and increase reach. In completing this Module, we recommend selecting the box corresponding to the primary goal of the modification or selecting multiple boxes in the context of several co-equal goals.

Second, we recommend documenting the level of the organization that most directly informed the modification. For example, modifications made to accommodate available staffing at a clinic would qualify as the organizational level, while modifications made to fit with the professional or cultural values of frontline staff delivering the EBP would qualify as the practitioner level.

### Module 5: When the modification is initiated, and whether it is planned

The timing of modifications is crucial in implementation science, with the pre-implementation, implementation, and sustainment phases featuring distinct pressures, challenges, and goals [37, 41, 42]. Modifications made early in the implementation process may leave more time for implementers and practitioners to adjust to the change. Modifications made later (during the implementation or sustainment phases) may nonetheless be required to accommodate shifting priorities or resources (e.g., shifting initial training to a web-based format based on travel restrictions). Documenting the timing of modifications to implementation strategies will allow the field to develop a better understanding of how such timing affects implementation processes and outcomes.

Note that for our purposes here the primary goal is to document when a modification is *initiated*, rather than when it *occurs*, as many modifications to implementation strategies may happen over large portions of the implementation period. For example, consider an implementation strategy of provider training to increase uptake of an evidence-based psychotherapy. If the timing of those trainings is modified, the important question for this section is *when the decision was made to change the timing* rather than when the (modified) trainings were offered. Documenting when decisions regarding modifications are made may ultimately help shed light on whether modifications made early versus late in the implementation period are differentially successful.

Regardless of the phase in which modifications to implementation strategies are made, there is a conceptual distinction between those that are made in a planful versus reactive manner [43, 44]. For example, consider a hypothetical implementation project featuring a learning collaborative that was originally designed to meet face-to-face four times during the implementation year. Let us further imagine that, due to budget constraints, only two learning collaborative meetings could be funded. If the budget constraints were known early in the implementation year, then the implementer and/or content experts would likely be able to modify the curriculum or format to ensure that all core content of the learning collaborative is covered (i.e., an adaptation or planned modification). Such proactive changes could include, for example, establishing or expanding a virtual component for the learning collaborative, or lengthening the two face-to-face sessions that could be funded. In contrast, having two face-to-face learning collaborative meetings abruptly canceled *midway through* the implementation year (i.e., a reactive modification) might necessitate more substantial changes to the curriculum that leaves some core content unaddressed. Differentiating unplanned, reactive modifications from proactive and planful adaptations will allow implementation scientists to better understand the circumstances under which impromptu modifications to implementation strategies may be more or less helpful.

### Module 6: Who participates in the decision to modify?

In some cases, modifications to implementation strategies may be made in a collaborative manner, with multiple stakeholders or “actors” (e.g., administrators, frontline clinicians, implementation specialists) agreeing that a given modification is needed in a given setting [16]. In other cases, the decision to modify an implementation strategy may be unilateral (as when a health system leader requires that a given implementation strategy be scaled back based on personnel changes or competing priorities). Documenting this distinction can inform future decisions regarding when broader consensus on certain types of modifications to implementation strategies is required for implementation success, consistent with the principles of stakeholder engagement [45]. As researchers start to grapple with the intersection of implementation science and health equity [46], carefully identifying who is suggesting the modifications may be an important aspect of tracking the co-creation of implementation strategies [21–24]. Identifying sources of power in the implementation process [47], and incorporating the voices of those in the community, will be important for the field as we move toward equitable practice in our science.

### Module 7: How widespread is the modification?

For modifications documented in Module 2 as Content, Training, and Evaluation modifications, it may be important to document the breadth or scope of the modification to the implementation strategy. This can range from relatively narrow modifications (e.g., in the context of an individual consultation call for a clinician who missed a day of group consultation) to broad-based ones (e.g., modifications made by an entire health system that is using an implementation strategy to roll out an EBP).

Note that some of the answer options refer to the individuals receiving the EBP, while others refer to the practitioners delivering the EBP, and yet others refer to those tasked with supporting the use of the EBP. It is possible that boxes within all three of these categories could be checked. If a single implementation facilitator adds an audit and feedback component to an implementation facilitation strategy within one clinic (and no other facilitators are using the unmodified strategy within the clinics they oversee), then that would qualify as a modification at one clinic/unit and one specific implementer/facilitator.

## Case example: Application of FRAME-IS in a recent implementation trial

Here, and in Table 2, we illustrate application of the FRAME-IS in an ongoing trial that seeks to train lay health workers to task-share with mental health professionals to improve engagement for Spanish-speaking Latinx families receiving Parent-Child Interaction Therapy (PCIT [48];). PCIT is an evidence-based parenting program for young children with disruptive behavior disorders [49]. In this task-sharing model, professional clinicians provide PCIT and lay health workers conduct outreach and promote treatment adherence to improve access, adherence, and skill acquisition for families [31]. Lay health workers have been identified as an important workforce to decrease disparities in access to care for marginalized communities [50]. However, limited research has identified what implementation supports lay health workers need to successfully engage in EBPs.

**Table 2:** Example completion of the FRAME-IS

FRAME-IS module or sub-component	Example completion
Module 1	
The EBP being implemented is:	Parent-Child Interaction Therapy (PCIT)
The implementation strategy being modified is:	Training program for lay health workers to enhance parent engagement in PCIT
The modification(s) being made is/are:	- Tailoring of training content (e.g., language) to local context to fit population differences - Removal of the behavioral coding component of the training
The reason(s) for the modification(s) is/are:	- Improve appropriateness/feasibility
Module 2	
What is modified?	- Content (details provided in Module 3) - Context (setting, based on transition from Miami to California)
Module 3	
What is the nature of the content, evaluation, or training modification?	- Tailoring (modifying language) - Removing/skipping elements (specifically, removal of behavioral coding training component)
OPTIONAL: what is the relationship to core elements?	- Unknown
Module 4	
What is the goal?	- Increase the acceptability, appropriateness, and feasibility of the implementation effort
What is the level of the rationale for the modification?	- Practitioner and Patient level (address cultural and linguistic differences for a population of predominantly Mexican descent)
Module 5	
When is the modification initiated?	- Pre-implementation/planning/pilot phase
Is the modification planned?	- Planned/proactive
Module 6	
Who participates in the decision to modify?	- Researcher, program leader, and clinicians (lay health workers)
OPTIONAL: Who makes the ultimate decision?	- Researchers
Module 7	
How widespread is the modification?	- Network system/community (listed modifications were applied for entire California rollout)

### Module 1: Description of the implementation strategy

In this case example, we will apply the FRAME-IS to the training model for lay health workers that was developed to support parental engagement in PCIT within a university-based clinic in Miami, Florida. Initial training aimed to prepare lay health workers to identify and refer appropriate cases to PCIT, teach them the parenting skills targeted in the program (e.g., giving specific praises of positive behaviors), and prepare them to promote adherence to home practice of these skills. Lay health workers were taught how to use a behavioral coding system in PCIT; they were also taught to provide feedback on how to improve weaker skills and reinforce skills that parents were using well. Each lay health worker was provided with an e-book with videos and scripts to help them describe PCIT and the parenting skills to caregivers. Lay health workers in the initial training demonstrated improved knowledge of PCIT over the course of training. They also increased their ability to model the parenting skills taught in PCIT, in order to help parents use them in the home. However, it was challenging for lay health workers to conduct behavioral observations of parent’s skill use and provide accurate feedback on which skills to improve [48].

### Module 2: What was modified?

The original training model was adapted for community-based mental health settings within California (context), with changes to the training program content to meet this context and address challenges identified in the initial training program evaluation.

### Module 3: The nature of the content modification

The majority of modifications involved *tailoring* (i.e., making minor changes to) the training materials to fit with the local context. Furthermore, the focus on training the lay health workers to conduct behavioral coding was *removed* from the training, as this was not considered a feasible or necessary skill. Instead, lay health workers were taught how to provide general feedback regarding parenting skills.

### Module 4: The rationale for the modification

The goal of the adaptations made was to increase the acceptability, appropriateness, and feasibility of the training to fit with cultural differences for providers and clients across the two contexts. Trainings in Miami and California were conducted in Spanish; however, regional language differences needed to be addressed. The Latinx population served in Miami was predominantly from the Caribbean (e.g., Cuba), South America (e.g., Colombia), and Central America (e.g., Honduras), whereas the population served in California was predominantly from Mexico. Therefore, certain vocabulary and idioms were tailored in training materials. New videos were created for the e-book, featuring children and parents of Mexican descent, to increase cultural and linguistic similarities with the families being served. Behavioral coding was removed from the training to increase the feasibility of training and to ensure adequate fidelity (skill at providing accurate feedback to the parents).

### Module 5: When the modification was initiated and planned

Modifications of the training protocol occurred during the pre-implementation/planning phase of the current trial and were planned as part of a research-community partnership (i.e., were proactively made prior to beginning training).

### Module 6: Who participated in decisions to modify?

Researchers, who developed and evaluated the initial training in Miami, led decisions surrounding the modifications to the training program based on data from the Miami project. They incorporated feedback from local program leaders and lay health workers, who participated in qualitative interviews and a community-advisory board. Ultimately, this feedback led to modifications that the research team finalized.

### Module 7: How widespread is the modification?

Modifications were made for the entire network system/community, specifically for lay health workers living in California and working within community-mental health settings.

## Discussion

Recent implementation science work has emphasized the importance of documenting modifications to EBPs [8, 10]. Implementation strategies are used to support the implementation of EBPs, and scholars have recommended specifying components of implementation strategies [16] to support their reproducibility and further elucidate mechanisms of change. As implementation strategies are deployed, however, they can be modified. Comprehensively documenting modifications to implementation strategies will allow the field to study the relationship between those modifications and implementation outcomes across settings. While previous frameworks (e.g., [10]) are meant to track modifications to EBPs, the FRAME-IS is to our knowledge the first tracking tool developed specifically for modifications to implementation strategies, incorporating modular coding and a novel focus on those implementing the EBP in question (see Supplemental File). Systematic application of the FRAME-IS to implementation projects—alongside careful assessment of implementation outcomes using RE-AIM [35] or similar evaluation-oriented implementation frameworks—will ideally help to answer fundamental questions about whether, when, how, and why an implementation strategy has been effective in a given setting. This will allow for a co-creation of collaborative knowledge between implementation researchers and stakeholders [21–24], as the FRAME-IS may guide systematic adaptations to implementation strategies when planning a project, and a way to track ad hoc modifications made throughout the project. The FRAME-IS may also be useful to test hypotheses related to core components or functions of implementation strategies: if a component of an implementation strategy is modified in some way, and that modified implementation strategy’s effectiveness rivals or surpasses that of its unmodified version, then it raises the possibility that the modified component (in its original form) was not in fact central to the implementation strategy’s success and that there are alternative, adaptive forms of the component that can be deployed successfully.

The FRAME-IS is not without limitations, of course. First, completing the FRAME-IS may be difficult in the context of subtle modifications that emerge longitudinally as implementation progresses (e.g., ongoing tailoring of written training materials), depending on who completes the reporting and how frequently it occurs. It may also be difficult to apply the FRAME-IS to multi-component implementation strategies (e.g., implementation facilitation [13]) that are inherently adaptable or intended to be tailored without guidance from the developers of the implementation strategy. In these circumstances, it may still represent a useful tool for tracking the specific ways that the implementation strategy was applied—albeit one that may require time-intensive multi-method assessment to achieve acceptable validity. It is also unclear whether local implementers are able to accurately report on modifications to implementation strategies as they occur in real time, or whether supplemental personnel are required. Local implementer reports may be necessary when expert observation is not feasible or scalable; in those situations, adequate training in the use of the FRAME-IS will be pivotal. One thing that remains to be determined is how frequently reporting should occur to capture the full extent of modifications. We also note that the FRAME-IS is meant to capture modifications to one *a priori* identified implementation strategy. Clear operationalization of what the original strategy entails is essential to accurately track adaptations. For projects featuring multiple implementation strategies rolled out over time (e.g., [51]), additional tracking (e.g., informed by the ERIC compilation [11] and with well-specified descriptions of the strategies as originally designed) may also be required to ensure a robust understanding not just of the modification process, but of how implementation proceeded more broadly.

### Conclusions

Implementation strategies often undergo modification to improve their fit with the EBPs, target populations, and clinical contexts in which they are applied. Without systematically tracking such modifications, it will be difficult for the implementation science field to determine how best to maximize their effectiveness or fully address health disparities [52]. To meet this need, the FRAME-IS is meant to be a first step toward better assessing the ways that implementation strategies may be modified as EBPs are put into practice. Fundamental questions, such as what role the FRAME-IS could play in designing, analyzing, and publishing implementation trials, will need to be explored in future work. Currently, the FRAME-IS is being piloted in a multinational ten-site implementation study; this and other applications of the FRAME-IS may help answer such questions regarding best practices for formatting, administering, and tracking its completion. It is our hope that application of the FRAME-IS more broadly may shed light on the processes and mechanisms by which implementation strategies exert their effects, ultimately improving the uptake of EBPs across settings.

## Supplementary Information

**Additional file 1.** This .ppt file contains supplemental descriptive text on the core and optional modules of the FRAME-IS.

## Data Availability

Not applicable

## Abbreviations

EBP: Evidence-Based Practice
FRAME: Framework for Reporting Adaptations and Modifications – Expanded
FRAME-IS: Framework for Reporting Adaptations and Modifications to Evidence-based Implementation Strategies
IRG: Implementation Research Group
PCIT: Parent-Child Interaction Therapy
RE-AIM: Reach – Effectiveness – Adoption – Implementation – Maintenance
WUNDIR: Washington University Network for Dissemination and Implementation Researchers
