# Supplementary material for: The FRAME-IS: a framework for documenting modifications to implementation strategies in healthcare
Source: Implement Sci. 2021 Apr 7;16:36. doi: 10.1186/s13012-021-01105-3 (PMC8024675; doi:10.1186/s13012-021-01105-3)
Supplement: Supplementary file 1 — Additional file 1. This .ppt file contains supplemental descriptive text on the core and optional modules of the FRAME-IS. [file 13012_2021_1105_MOESM1_ESM.pptx]

## Slide 1
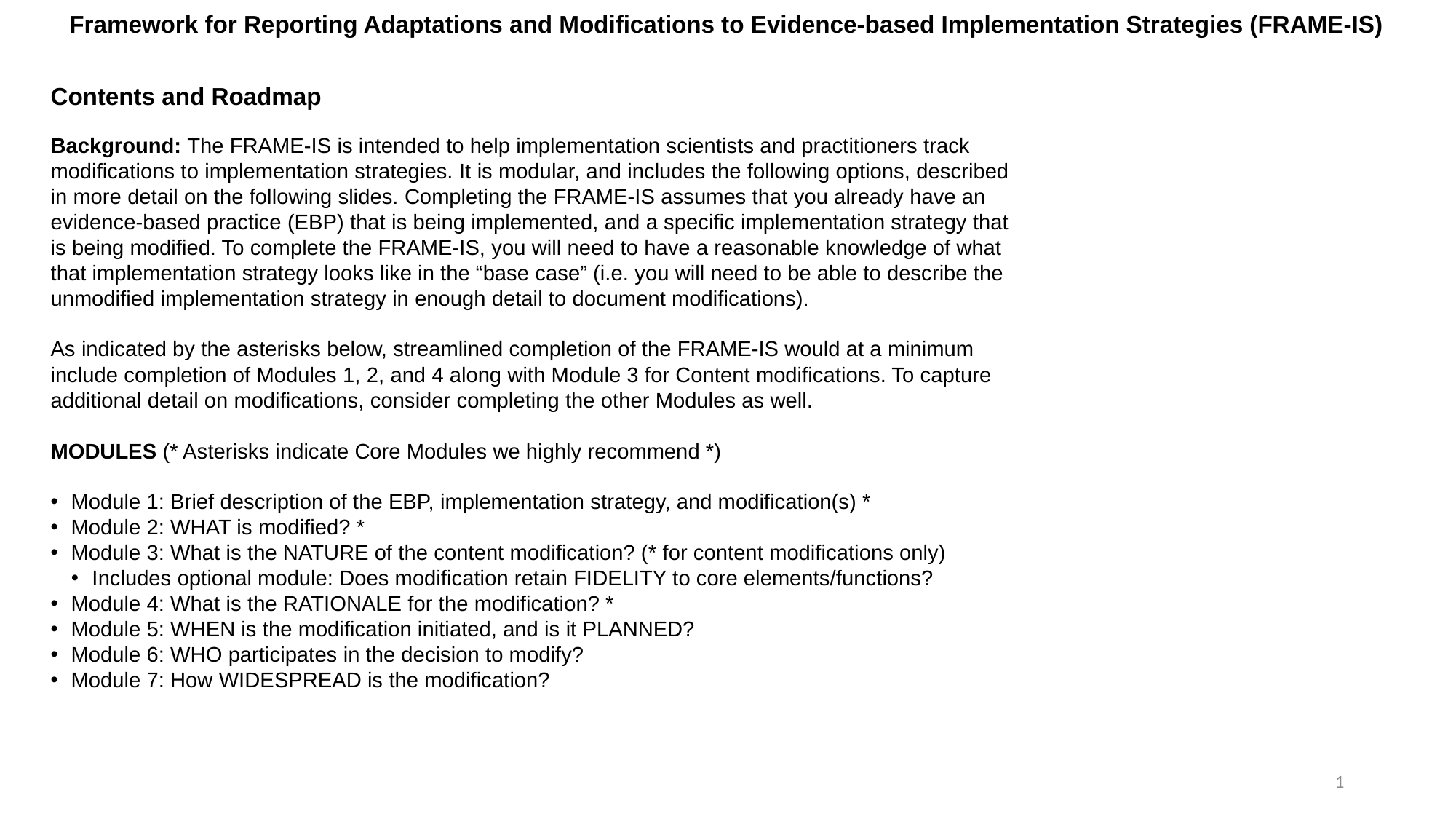

Contents and Roadmap
Background: The FRAME-IS is intended to help implementation scientists and practitioners track modifications to implementation strategies. It is modular, and includes the following options, described in more detail on the following slides. Completing the FRAME-IS assumes that you already have an evidence-based practice (EBP) that is being implemented, and a specific implementation strategy that is being modified. To complete the FRAME-IS, you will need to have a reasonable knowledge of what that implementation strategy looks like in the “base case” (i.e. you will need to be able to describe the unmodified implementation strategy in enough detail to document modifications).
As indicated by the asterisks below, streamlined completion of the FRAME-IS would at a minimum include completion of Modules 1, 2, and 4 along with Module 3 for Content modifications. To capture additional detail on modifications, consider completing the other Modules as well.
MODULES (* Asterisks indicate Core Modules we highly recommend *)
Module 1: Brief description of the EBP, implementation strategy, and modification(s) *
Module 2: WHAT is modified? *
Module 3: What is the NATURE of the content modification? (* for content modifications only)
Includes optional module: Does modification retain FIDELITY to core elements/functions?
Module 4: What is the RATIONALE for the modification? *
Module 5: WHEN is the modification initiated, and is it PLANNED?
Module 6: WHO participates in the decision to modify?
Module 7: How WIDESPREAD is the modification?
0

## Slide 2
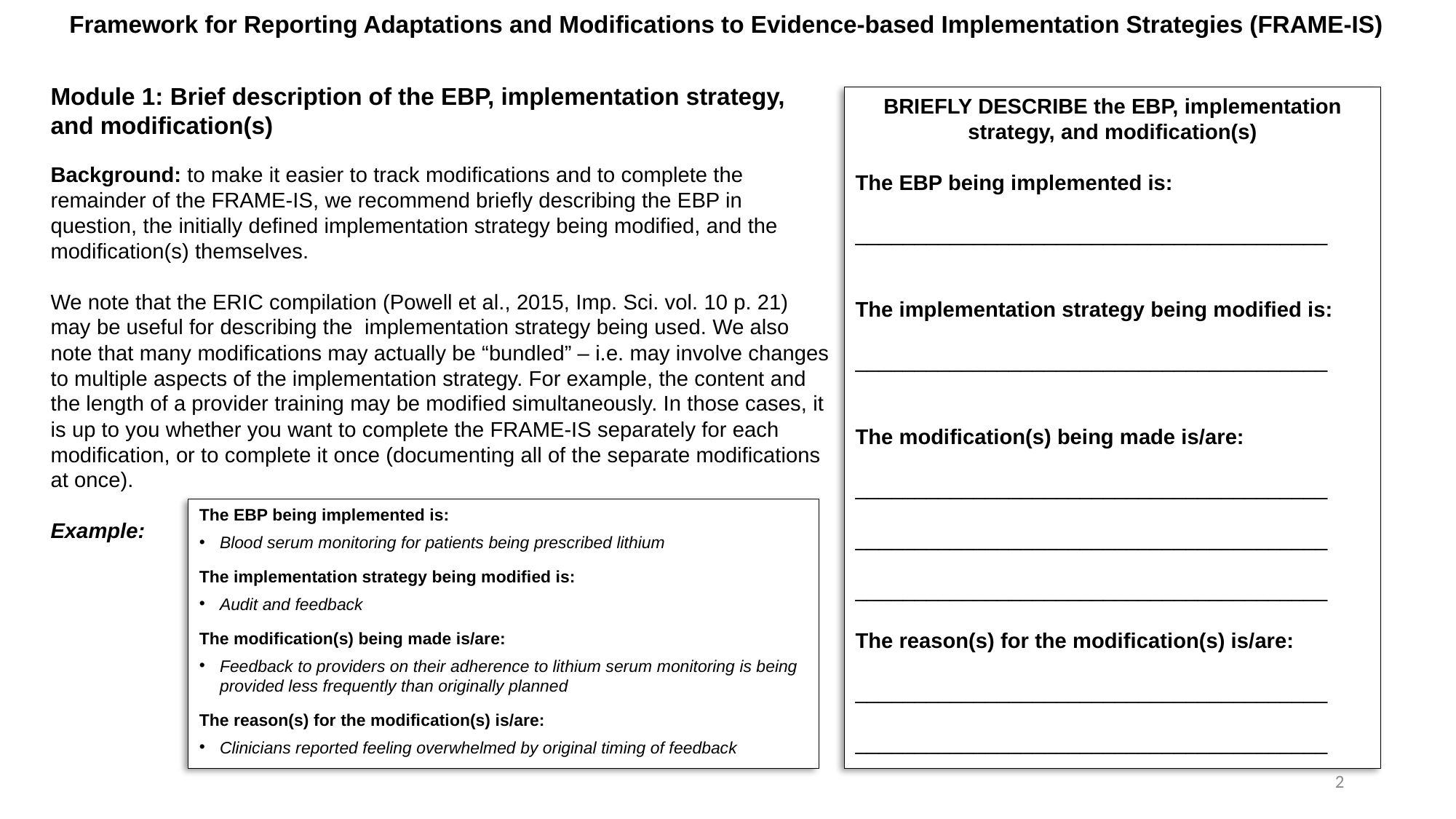

Module 1: Brief description of the EBP, implementation strategy, and modification(s)
Background: to make it easier to track modifications and to complete the remainder of the FRAME-IS, we recommend briefly describing the EBP in question, the initially defined implementation strategy being modified, and the modification(s) themselves.
We note that the ERIC compilation (Powell et al., 2015, Imp. Sci. vol. 10 p. 21) may be useful for describing the implementation strategy being used. We also note that many modifications may actually be “bundled” – i.e. may involve changes to multiple aspects of the implementation strategy. For example, the content and the length of a provider training may be modified simultaneously. In those cases, it is up to you whether you want to complete the FRAME-IS separately for each modification, or to complete it once (documenting all of the separate modifications at once).
Example:
BRIEFLY DESCRIBE the EBP, implementation strategy, and modification(s)
The EBP being implemented is:
________________________________________
The implementation strategy being modified is:
________________________________________
The modification(s) being made is/are:
________________________________________
________________________________________
________________________________________
The reason(s) for the modification(s) is/are:
________________________________________
________________________________________
The EBP being implemented is:
Blood serum monitoring for patients being prescribed lithium
The implementation strategy being modified is:
Audit and feedback
The modification(s) being made is/are:
Feedback to providers on their adherence to lithium serum monitoring is being provided less frequently than originally planned
The reason(s) for the modification(s) is/are:
Clinicians reported feeling overwhelmed by original timing of feedback
1

## Slide 3
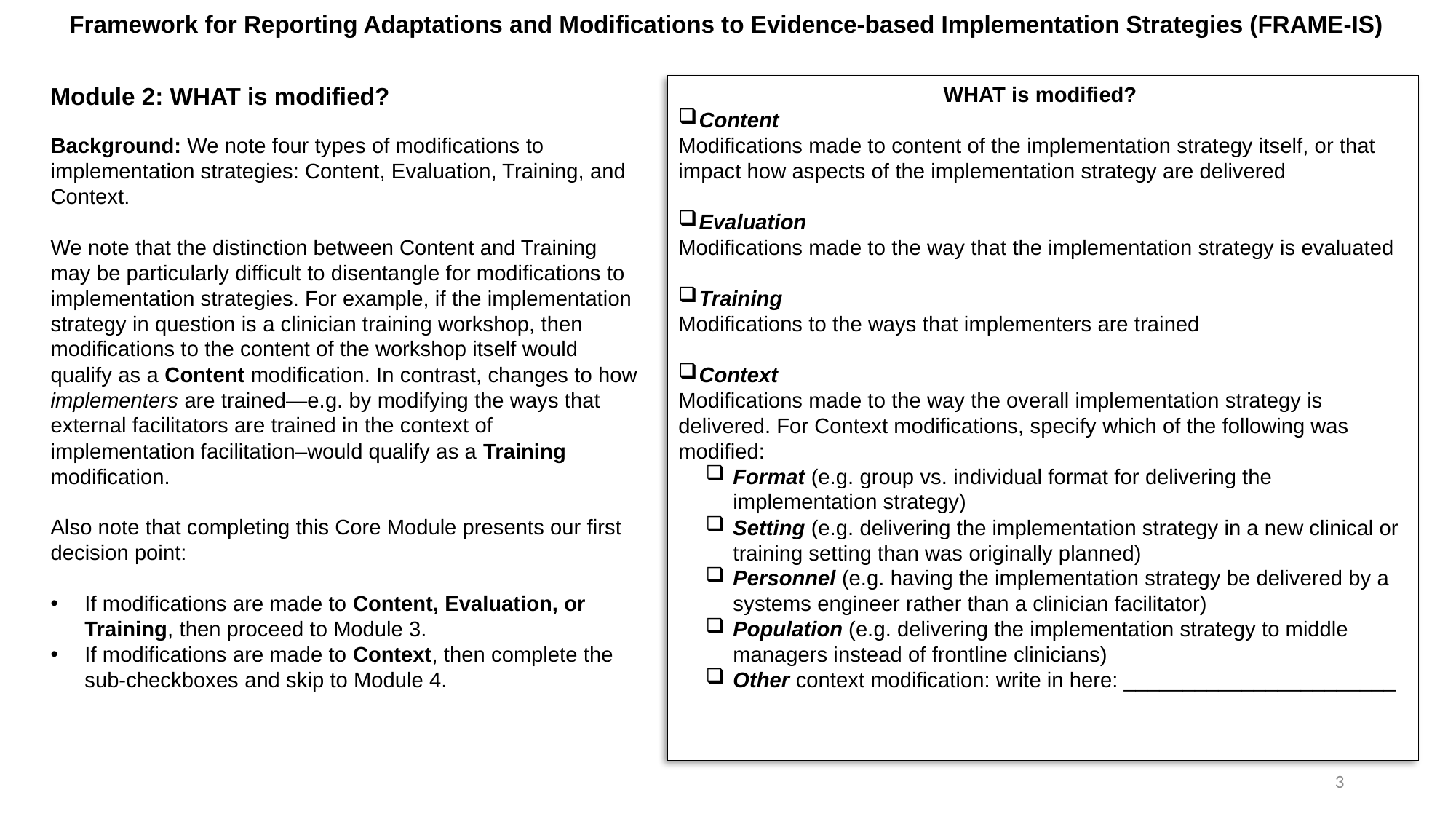

Module 2: WHAT is modified?
Background: We note four types of modifications to implementation strategies: Content, Evaluation, Training, and Context.
We note that the distinction between Content and Training may be particularly difficult to disentangle for modifications to implementation strategies. For example, if the implementation strategy in question is a clinician training workshop, then modifications to the content of the workshop itself would qualify as a Content modification. In contrast, changes to how implementers are trained—e.g. by modifying the ways that external facilitators are trained in the context of implementation facilitation–would qualify as a Training modification.
Also note that completing this Core Module presents our first decision point:
If modifications are made to Content, Evaluation, or Training, then proceed to Module 3.
If modifications are made to Context, then complete the sub-checkboxes and skip to Module 4.
WHAT is modified?
Content
Modifications made to content of the implementation strategy itself, or that impact how aspects of the implementation strategy are delivered
Evaluation
Modifications made to the way that the implementation strategy is evaluated
Training
Modifications to the ways that implementers are trained
Context
Modifications made to the way the overall implementation strategy is delivered. For Context modifications, specify which of the following was modified:
Format (e.g. group vs. individual format for delivering the implementation strategy)
Setting (e.g. delivering the implementation strategy in a new clinical or training setting than was originally planned)
Personnel (e.g. having the implementation strategy be delivered by a systems engineer rather than a clinician facilitator)
Population (e.g. delivering the implementation strategy to middle managers instead of frontline clinicians)
Other context modification: write in here: _______________________
2

## Slide 4
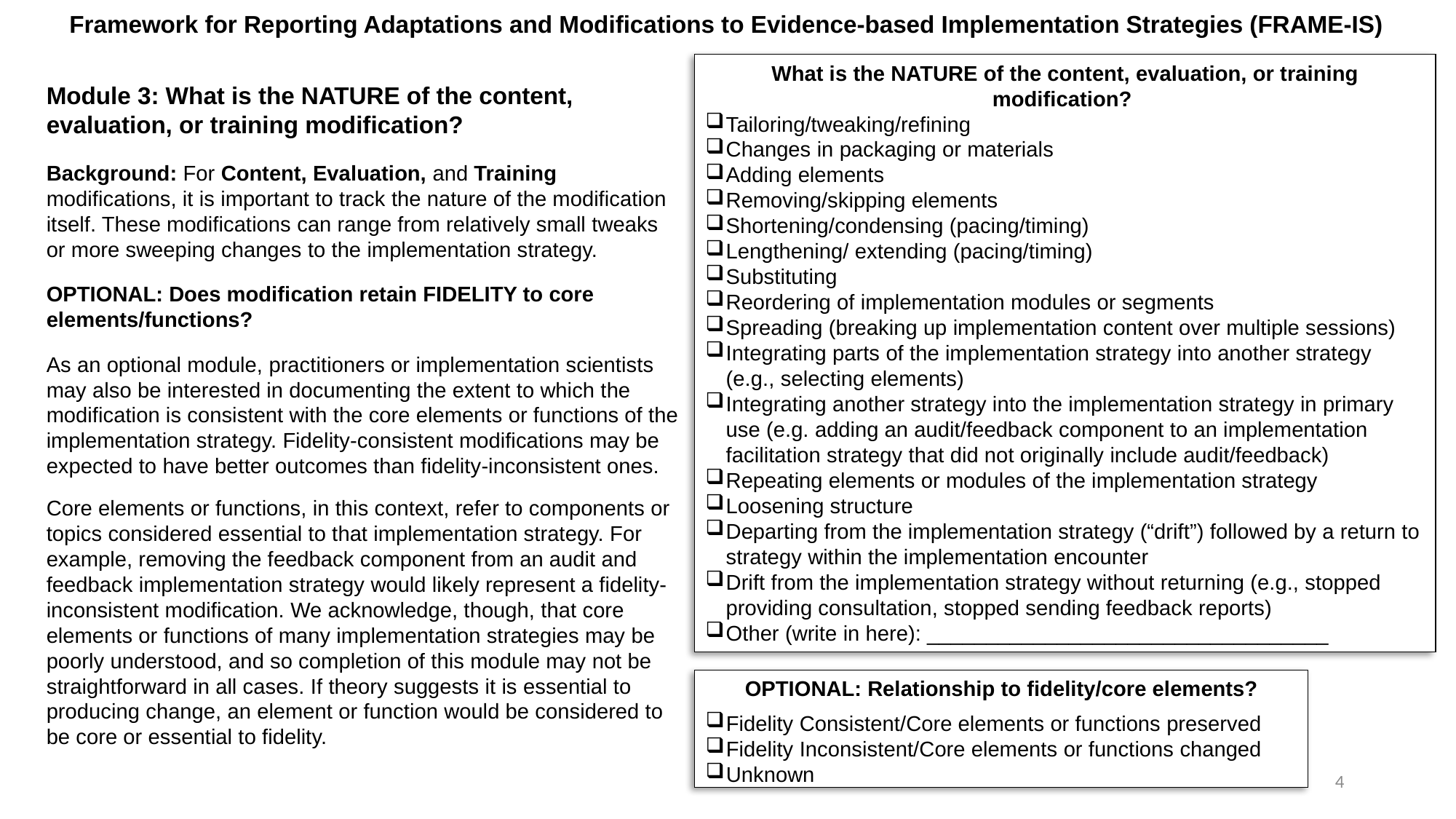

What is the NATURE of the content, evaluation, or training modification?
Tailoring/tweaking/refining
Changes in packaging or materials
Adding elements
Removing/skipping elements
Shortening/condensing (pacing/timing)
Lengthening/ extending (pacing/timing)
Substituting
Reordering of implementation modules or segments
Spreading (breaking up implementation content over multiple sessions)
Integrating parts of the implementation strategy into another strategy (e.g., selecting elements)
Integrating another strategy into the implementation strategy in primary use (e.g. adding an audit/feedback component to an implementation facilitation strategy that did not originally include audit/feedback)
Repeating elements or modules of the implementation strategy
Loosening structure
Departing from the implementation strategy (“drift”) followed by a return to strategy within the implementation encounter
Drift from the implementation strategy without returning (e.g., stopped providing consultation, stopped sending feedback reports)
Other (write in here): __________________________________
Module 3: What is the NATURE of the content, evaluation, or training modification?
Background: For Content, Evaluation, and Training modifications, it is important to track the nature of the modification itself. These modifications can range from relatively small tweaks or more sweeping changes to the implementation strategy.
OPTIONAL: Does modification retain FIDELITY to core elements/functions?
As an optional module, practitioners or implementation scientists may also be interested in documenting the extent to which the modification is consistent with the core elements or functions of the implementation strategy. Fidelity-consistent modifications may be expected to have better outcomes than fidelity-inconsistent ones.
Core elements or functions, in this context, refer to components or topics considered essential to that implementation strategy. For example, removing the feedback component from an audit and feedback implementation strategy would likely represent a fidelity-inconsistent modification. We acknowledge, though, that core elements or functions of many implementation strategies may be poorly understood, and so completion of this module may not be straightforward in all cases. If theory suggests it is essential to producing change, an element or function would be considered to be core or essential to fidelity.
OPTIONAL: Relationship to fidelity/core elements?
Fidelity Consistent/Core elements or functions preserved
Fidelity Inconsistent/Core elements or functions changed
Unknown
3

## Slide 5
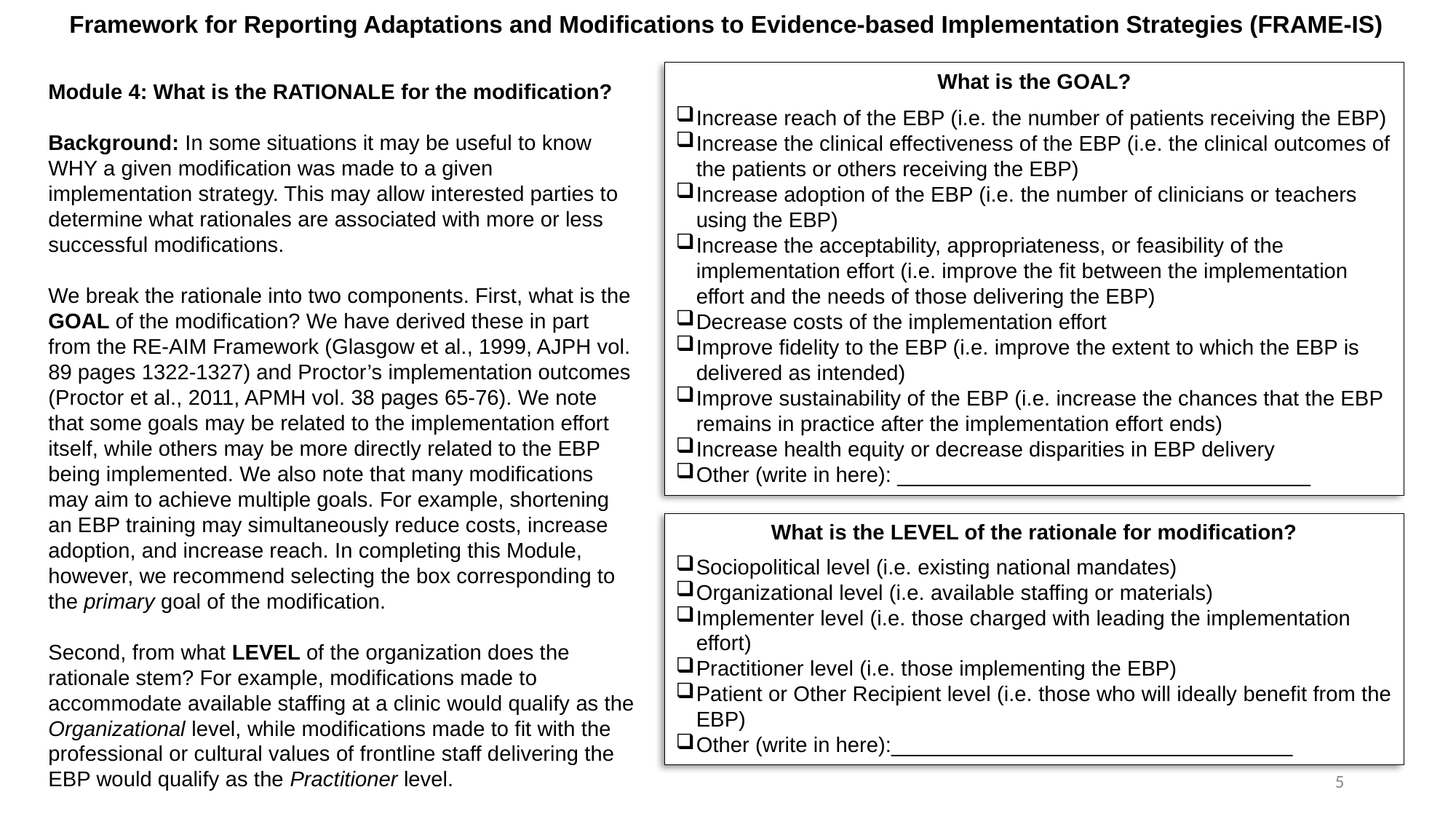

What is the GOAL?
Increase reach of the EBP (i.e. the number of patients receiving the EBP)
Increase the clinical effectiveness of the EBP (i.e. the clinical outcomes of the patients or others receiving the EBP)
Increase adoption of the EBP (i.e. the number of clinicians or teachers using the EBP)
Increase the acceptability, appropriateness, or feasibility of the implementation effort (i.e. improve the fit between the implementation effort and the needs of those delivering the EBP)
Decrease costs of the implementation effort
Improve fidelity to the EBP (i.e. improve the extent to which the EBP is delivered as intended)
Improve sustainability of the EBP (i.e. increase the chances that the EBP remains in practice after the implementation effort ends)
Increase health equity or decrease disparities in EBP delivery
Other (write in here): ___________________________________
Module 4: What is the RATIONALE for the modification?
Background: In some situations it may be useful to know WHY a given modification was made to a given implementation strategy. This may allow interested parties to determine what rationales are associated with more or less successful modifications.
We break the rationale into two components. First, what is the GOAL of the modification? We have derived these in part from the RE-AIM Framework (Glasgow et al., 1999, AJPH vol. 89 pages 1322-1327) and Proctor’s implementation outcomes (Proctor et al., 2011, APMH vol. 38 pages 65-76). We note that some goals may be related to the implementation effort itself, while others may be more directly related to the EBP being implemented. We also note that many modifications may aim to achieve multiple goals. For example, shortening an EBP training may simultaneously reduce costs, increase adoption, and increase reach. In completing this Module, however, we recommend selecting the box corresponding to the primary goal of the modification.
Second, from what LEVEL of the organization does the rationale stem? For example, modifications made to accommodate available staffing at a clinic would qualify as the Organizational level, while modifications made to fit with the professional or cultural values of frontline staff delivering the EBP would qualify as the Practitioner level.
What is the LEVEL of the rationale for modification?
Sociopolitical level (i.e. existing national mandates)
Organizational level (i.e. available staffing or materials)
Implementer level (i.e. those charged with leading the implementation effort)
Practitioner level (i.e. those implementing the EBP)
Patient or Other Recipient level (i.e. those who will ideally benefit from the EBP)
Other (write in here):__________________________________
4

## Slide 6
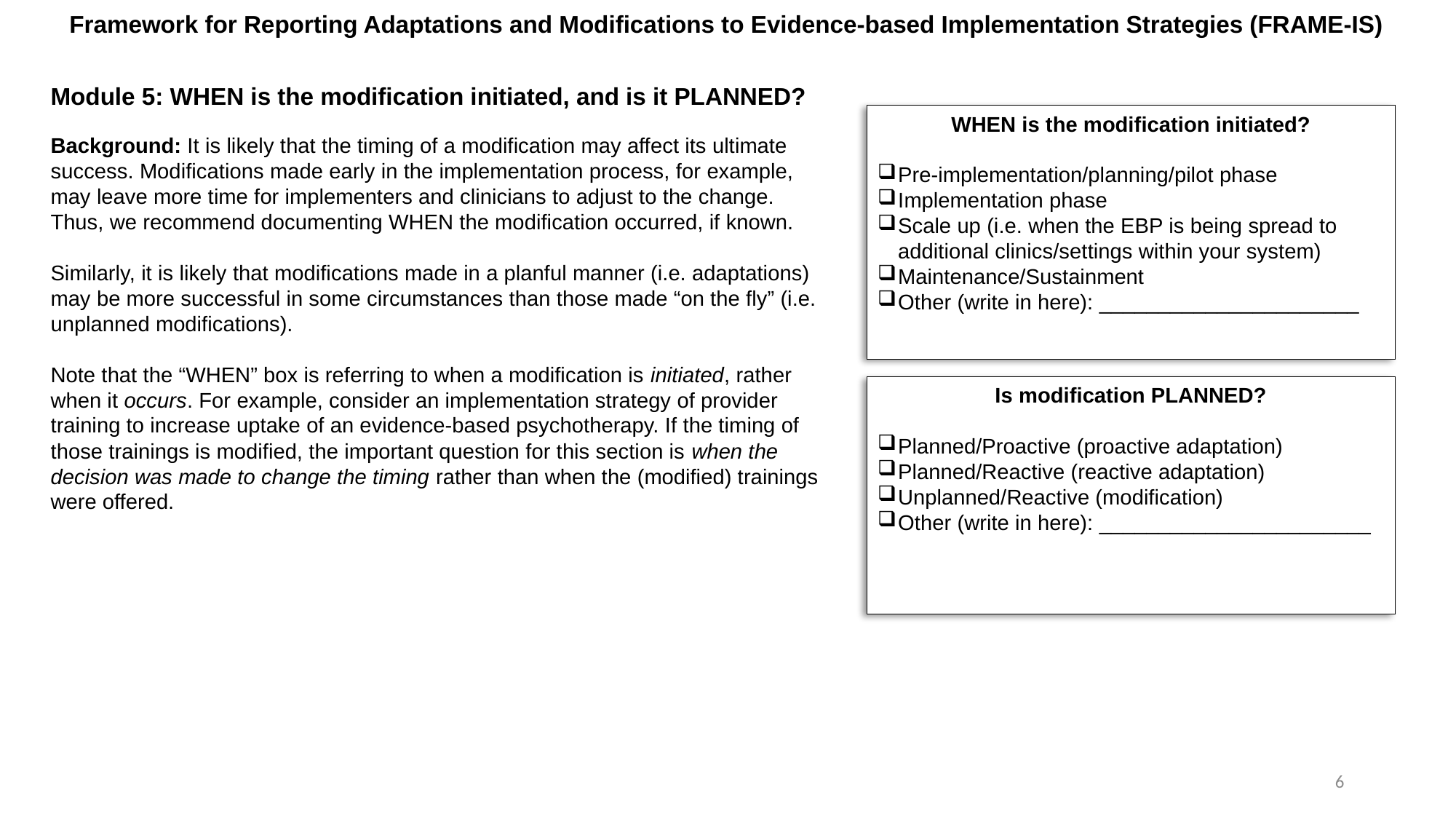

Module 5: WHEN is the modification initiated, and is it PLANNED?
Background: It is likely that the timing of a modification may affect its ultimate success. Modifications made early in the implementation process, for example, may leave more time for implementers and clinicians to adjust to the change. Thus, we recommend documenting WHEN the modification occurred, if known.
Similarly, it is likely that modifications made in a planful manner (i.e. adaptations) may be more successful in some circumstances than those made “on the fly” (i.e. unplanned modifications).
Note that the “WHEN” box is referring to when a modification is initiated, rather when it occurs. For example, consider an implementation strategy of provider training to increase uptake of an evidence-based psychotherapy. If the timing of those trainings is modified, the important question for this section is when the decision was made to change the timing rather than when the (modified) trainings were offered.
WHEN is the modification initiated?
Pre-implementation/planning/pilot phase
Implementation phase
Scale up (i.e. when the EBP is being spread to additional clinics/settings within your system)
Maintenance/Sustainment
Other (write in here): ______________________
Is modification PLANNED?
Planned/Proactive (proactive adaptation)
Planned/Reactive (reactive adaptation)
Unplanned/Reactive (modification)
Other (write in here): _______________________
5

## Slide 7
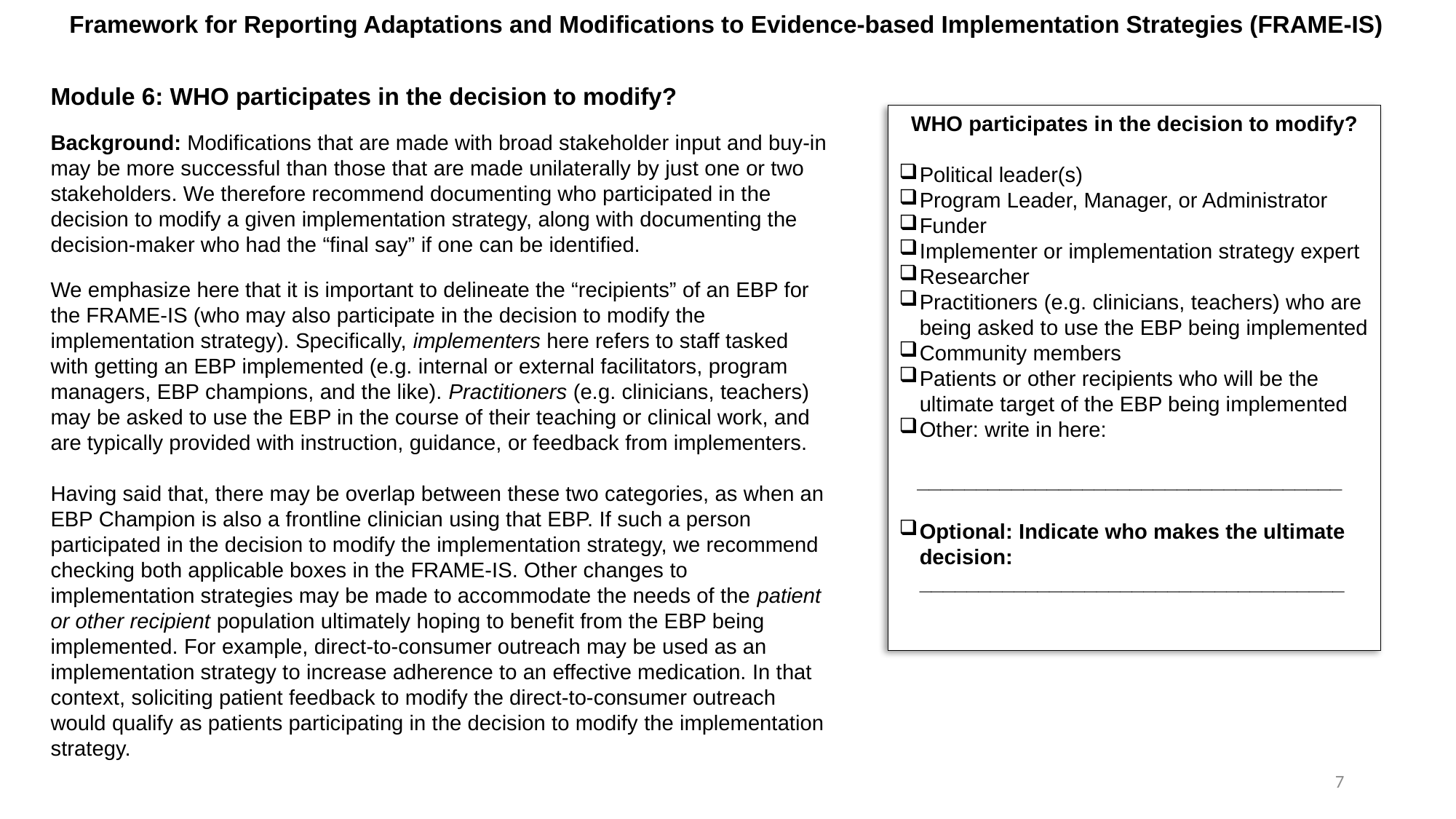

Module 6: WHO participates in the decision to modify?
Background: Modifications that are made with broad stakeholder input and buy-in may be more successful than those that are made unilaterally by just one or two stakeholders. We therefore recommend documenting who participated in the decision to modify a given implementation strategy, along with documenting the decision-maker who had the “final say” if one can be identified.
We emphasize here that it is important to delineate the “recipients” of an EBP for the FRAME-IS (who may also participate in the decision to modify the implementation strategy). Specifically, implementers here refers to staff tasked with getting an EBP implemented (e.g. internal or external facilitators, program managers, EBP champions, and the like). Practitioners (e.g. clinicians, teachers) may be asked to use the EBP in the course of their teaching or clinical work, and are typically provided with instruction, guidance, or feedback from implementers.
Having said that, there may be overlap between these two categories, as when an EBP Champion is also a frontline clinician using that EBP. If such a person participated in the decision to modify the implementation strategy, we recommend checking both applicable boxes in the FRAME-IS. Other changes to implementation strategies may be made to accommodate the needs of the patient or other recipient population ultimately hoping to benefit from the EBP being implemented. For example, direct-to-consumer outreach may be used as an implementation strategy to increase adherence to an effective medication. In that context, soliciting patient feedback to modify the direct-to-consumer outreach would qualify as patients participating in the decision to modify the implementation strategy.
WHO participates in the decision to modify?
Political leader(s)
Program Leader, Manager, or Administrator
Funder
Implementer or implementation strategy expert
Researcher
Practitioners (e.g. clinicians, teachers) who are being asked to use the EBP being implemented
Community members
Patients or other recipients who will be the ultimate target of the EBP being implemented
Other: write in here:
 ____________________________________
Optional: Indicate who makes the ultimate decision: ____________________________________
6

## Slide 8
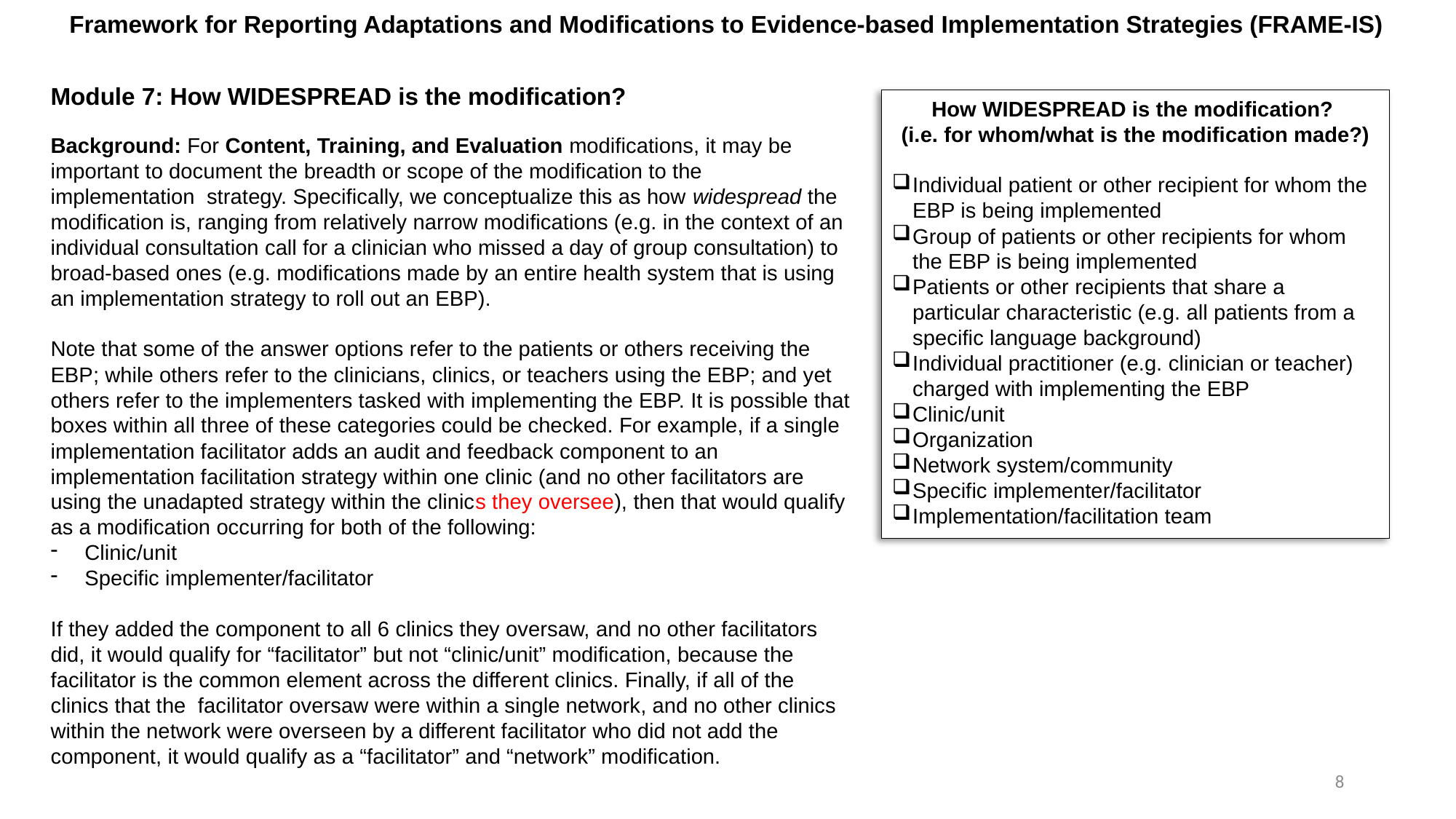

Module 7: How WIDESPREAD is the modification?
Background: For Content, Training, and Evaluation modifications, it may be important to document the breadth or scope of the modification to the implementation strategy. Specifically, we conceptualize this as how widespread the modification is, ranging from relatively narrow modifications (e.g. in the context of an individual consultation call for a clinician who missed a day of group consultation) to broad-based ones (e.g. modifications made by an entire health system that is using an implementation strategy to roll out an EBP).
Note that some of the answer options refer to the patients or others receiving the EBP; while others refer to the clinicians, clinics, or teachers using the EBP; and yet others refer to the implementers tasked with implementing the EBP. It is possible that boxes within all three of these categories could be checked. For example, if a single implementation facilitator adds an audit and feedback component to an implementation facilitation strategy within one clinic (and no other facilitators are using the unadapted strategy within the clinics they oversee), then that would qualify as a modification occurring for both of the following:
Clinic/unit
Specific implementer/facilitator
If they added the component to all 6 clinics they oversaw, and no other facilitators did, it would qualify for “facilitator” but not “clinic/unit” modification, because the facilitator is the common element across the different clinics. Finally, if all of the clinics that the facilitator oversaw were within a single network, and no other clinics within the network were overseen by a different facilitator who did not add the component, it would qualify as a “facilitator” and “network” modification.
How WIDESPREAD is the modification?
(i.e. for whom/what is the modification made?)
Individual patient or other recipient for whom the EBP is being implemented
Group of patients or other recipients for whom the EBP is being implemented
Patients or other recipients that share a particular characteristic (e.g. all patients from a specific language background)
Individual practitioner (e.g. clinician or teacher) charged with implementing the EBP
Clinic/unit
Organization
Network system/community
Specific implementer/facilitator
Implementation/facilitation team
7
